# Medical Items

**Published:** 1895-11

**Authors:** 


### *MEDICAL ITEMS.*

Dr. H. N. Hollifield, of Sandersville, Ga., died September 24th, aged 63.

The office of The Journal is now in room 311 Fitten building, corner of Broad and Marietta streets.

Dr. Miller B. Hutchins has changed his office from the Equitable building to Nos. 311 and 312 Fitten building.

We have received the first number of a new journal published in the interest of a mudbath sanatorium in Indiana. Its name is *Mud*.

Dr. William J. Finley, of Charleston, S. C., has been selected by the sanitary board of Savannah to fill the vacancy as quarantine officer left by the resignation of Dr. Joseph B. Graham.

A subscriber in Temperance, Ga., writes us: "I like your journal. It has been a great help to me, and is certainly the cleanest and squarest medical publication I have ever read." Thanks. That is the kind of journal we are trying to make.

It is a mistake to pay so much attention to pathology and the causes of disease that therapeutics must be neglected. From the point of view of the invalid, it is infinitely more important to know the remedy than it is to know the microbic or other cause of his disease.—*Ex.*

Another victim to "Christian Science."—Mr. V. H. Hitt, of Americus, was taken ill with what was supposed to be hemorrhagic fever. Some friends called in a few "Christian Science" fanatics, and none but these were allowed to see the sick man. He promptly died, a victim to neglect and a delusion.

MR. W. B. SAUNDERS, publisher (925 Walnut St., Philadelphia), announces the preparation of the "American Year-Book of Medicine," edited by Dr. Geo. M. Gould. The book will be a practical report of medical progress throughout the world, including only what is new. It will be ready for delivery January 1st.

---

IN view of the fatal results sometimes following the sewing up of sponges, forceps, scissors, pads, etc., in the abdomen, an exchange calls attention to the importance of a thorough count of all the articles of the operative paraphernalia, including the assistants and nurses; one of the latter might be left in the abdominal cavity.

DOCTOR, don't, for Heaven's sake, daub a lot of "black salve" or any other kind of salve or vaseline, ointment or liniment—no matter what its nature, upon a *fresh cut.* Instead of putting *foreign substance* into it or about it, remove all such—cleanse it thoroughly, then tie it up neatly in its own blood and let nature, God's handmaid, do the rest. It will be done and well done. Of course if necessary put in a stitch or two with clean needle and silk, and clean hands.—*Medical Gleaner.*

---

MEDICAL etiquette is a sealed book to the laity, and yet when a judge, in endeavoring to settle a dispute on ethics between two physicians, said that he could not make much out of medical etiquette, but if the two physicians would go home and behave like gentlemen he thought that the trouble would be cleared up, he probably understood the matter. If a man is a gentleman in the correct meaning of the word, and acts to others as he would have them act towards him, he will need no medical etiquette.—*Maryland Medical Journal.*

---

ERRATA.—We regret to note some errors in Dr. Allen's article on "Pneumothorax" in our September issue. On page 393, second line, read *different* for *difficult;* same page, second line from bottom, read *bronchi* for *branches;* page 394, fourth line, read *spot* for *sprout;* same page, eleventh line from bottom, period should be placed after *increase*, and the semicolon omitted after *present;* page 396, eighteenth line, *reputed* should be *reported;* and page 398, last line, *systematically* should be *symptomatically.* Our proof-reader will be more careful in the future.

---

THE Southern Surgical and Gynecological Association will hold its eighth annual meeting at Washington, D. C., November 12, 13 and 14. The secretary, Dr. W. E. B. Davis, of Birmingham, has prepared a program replete with interesting titles by able authors, which bespeaks an instructive meeting. The following named are the officers elected for this meeting: President, Dr. Louis McLane Tiffany, of Baltimore; vice-presidents, Dr. Ernest S. Lewis, of New Orleans, and Dr. Manning Simons, of Charleston; treasurer, Dr. Richard Douglas, of Nashville; secretary, Dr. W. E. B. Davis, of Birmingham.

